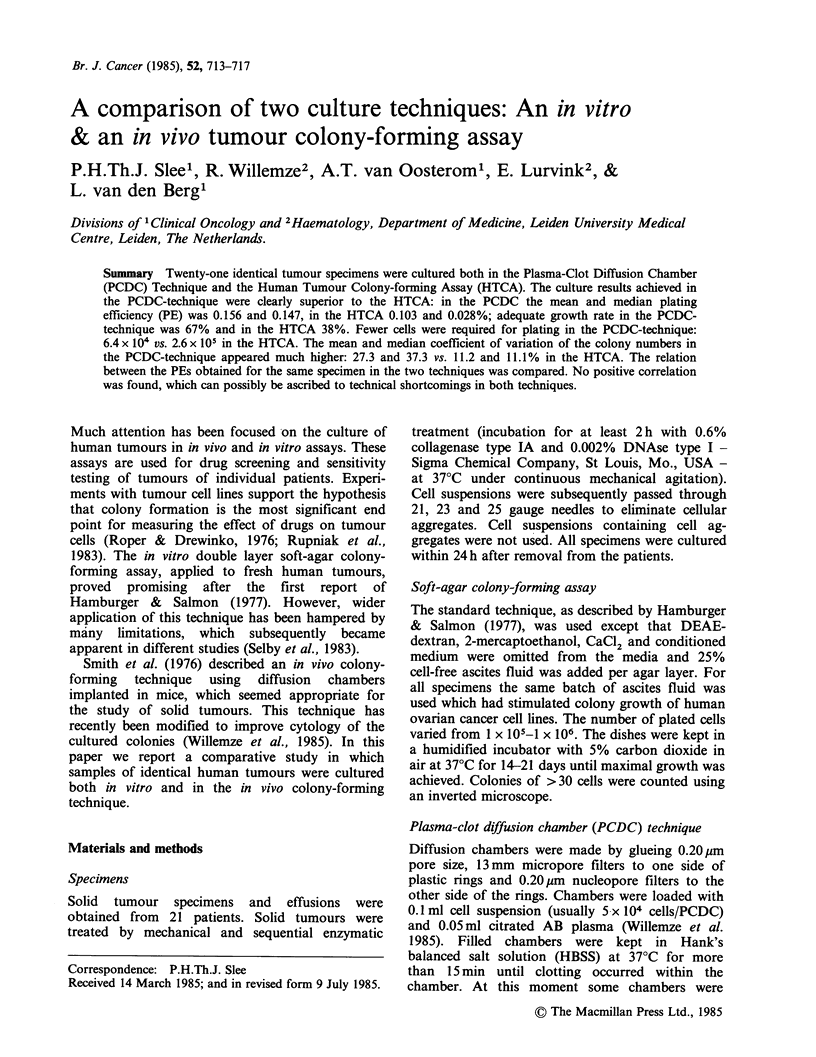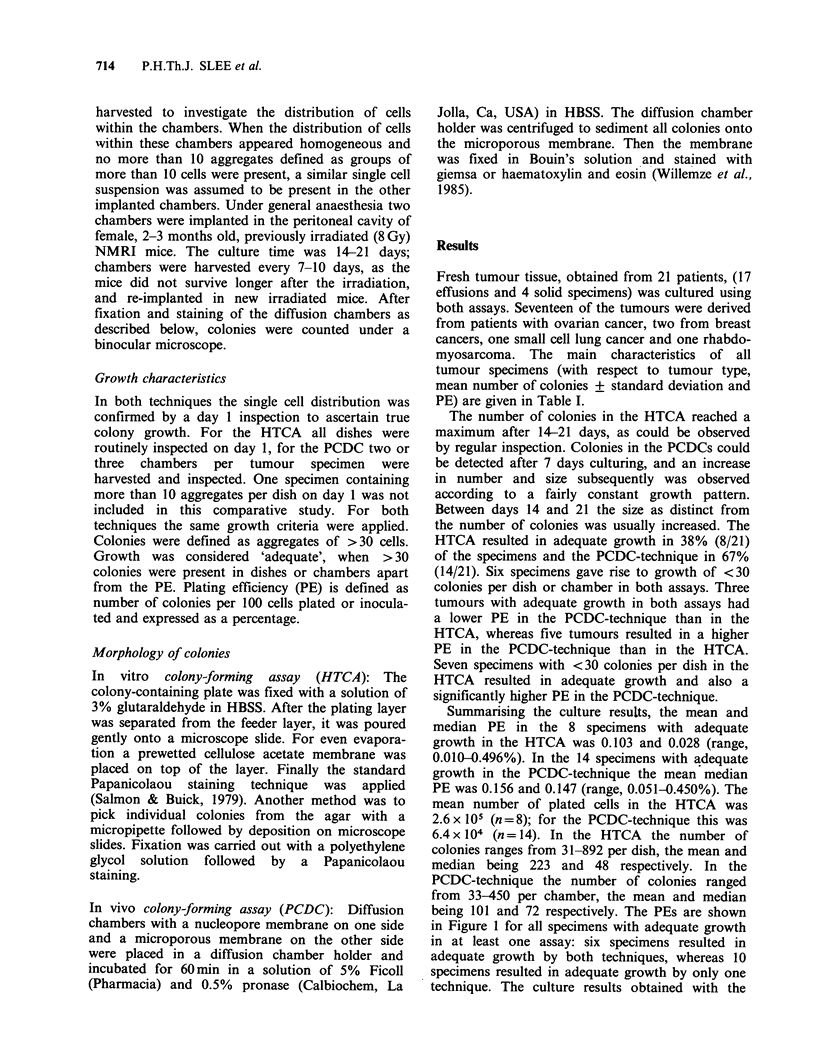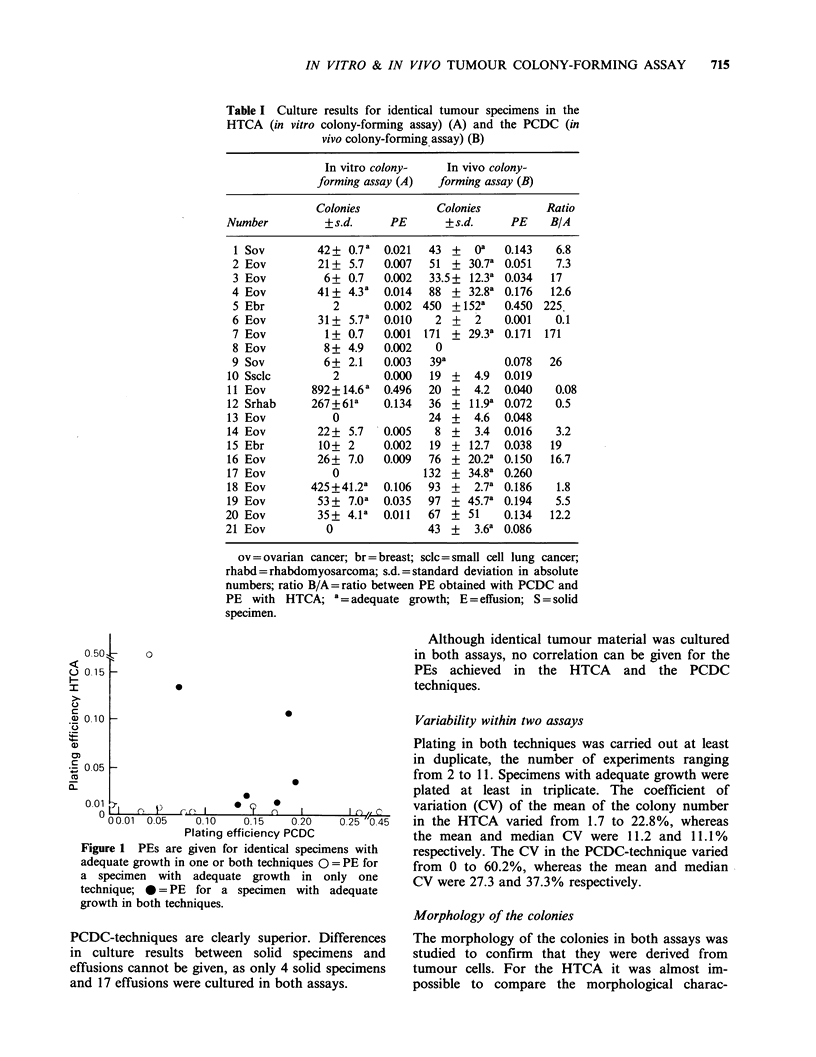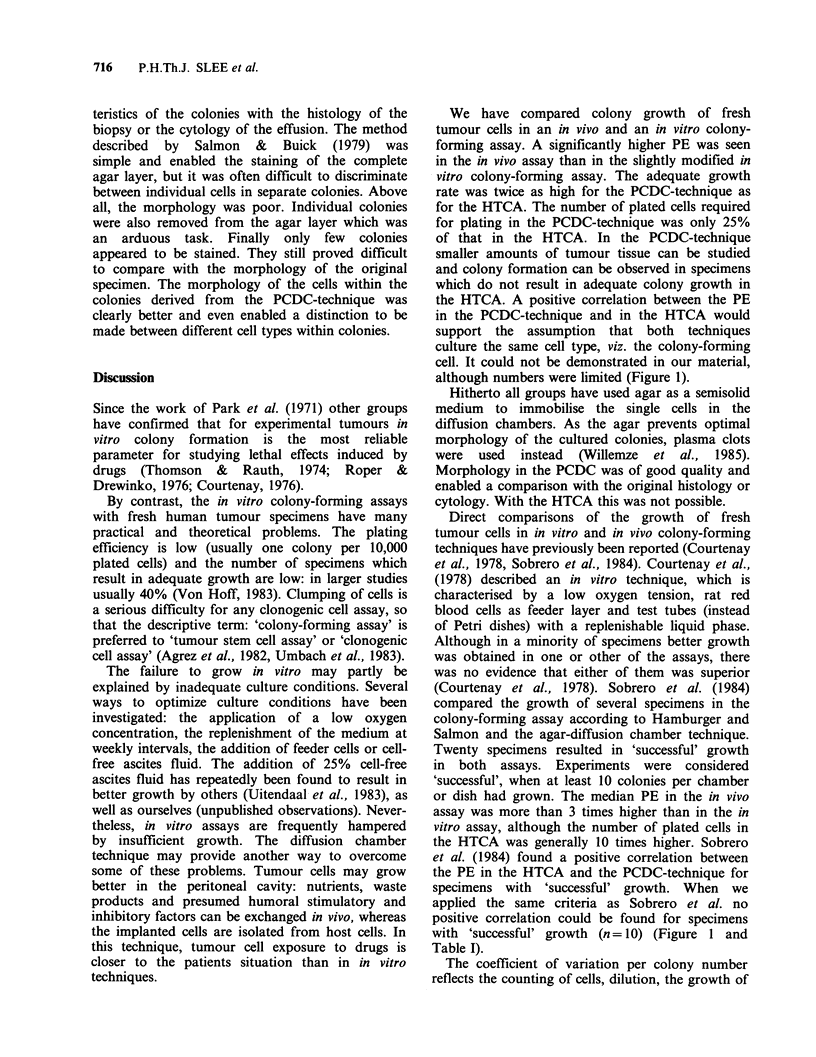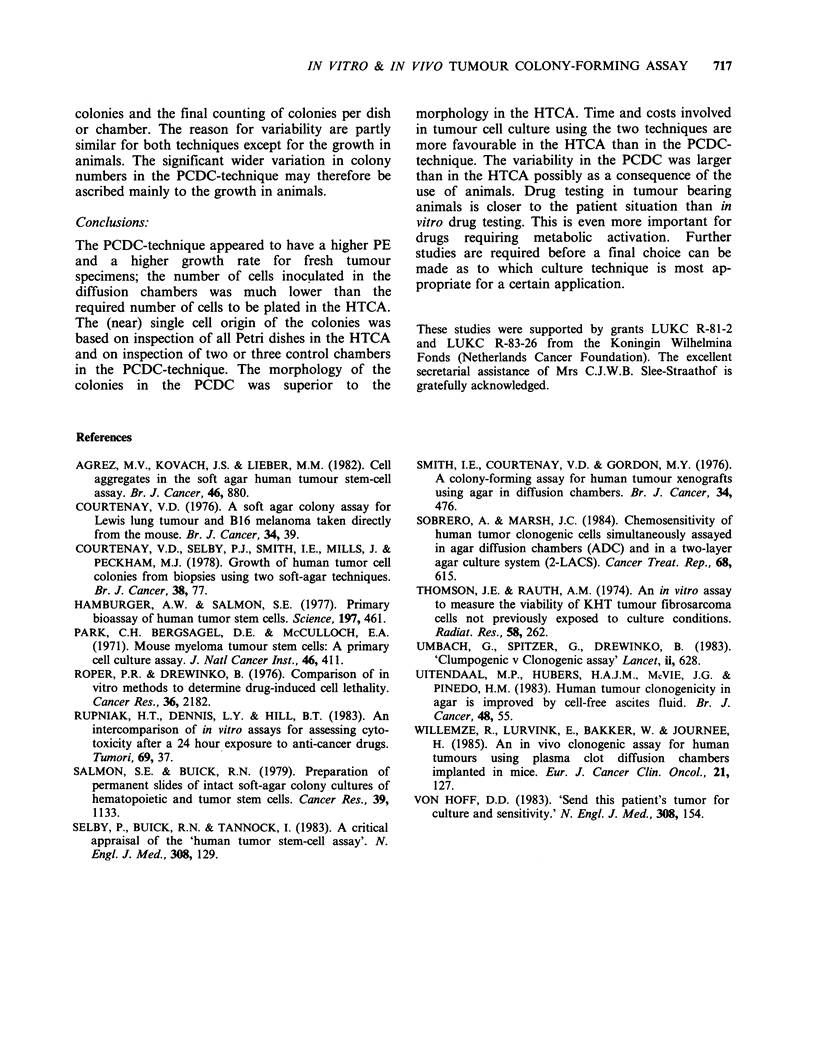# A comparison of two culture techniques: an in vitro & an in vivo tumour colony-forming assay.

**DOI:** 10.1038/bjc.1985.248

**Published:** 1985-11

**Authors:** P. H. Slee, R. Willemze, A. T. van Oosterom, E. Lurvink, L. van den Berg

## Abstract

Twenty-one identical tumour specimens were cultured both in the Plasma-Clot Diffusion Chamber (PCDC) Technique and the Human Tumour Colony-forming Assay (HTCA). The culture results achieved in the PCDC-technique were clearly superior to the HTCA: in the PCDC the mean and median plating efficiency (PE) was 0.156 and 0.147, in the HTCA 0.103 and 0.028%; adequate growth rate in the PCDC-technique was 67% and in the HTCA 38%. Fewer cells were required for plating in the PCDC-technique: 6.4 X 10(4) vs. 2.6 X 10(5) in the HTCA. The mean and median coefficient of variation of the colony numbers in the PCDC-technique appeared much higher: 27.3 and 37.3 vs. 11.2 and 11.1% in the HTCA. The relation between the PEs obtained for the same specimen in the two techniques was compared. No positive correlation was found, which can possibly be ascribed to technical shortcomings in both techniques.


*Br. J. Cancer* (1985), **52**, 713–717

### A comparison of two culture techniques: An *in vitro* & an *in vivo* tumour colony-forming assay

P.H.Th.J. Slee[1], R. Willemze[2], A.T. van Oosterom[1], E. Lurvink[2], & L. van den Berg[1]

*Divisions of [1]Clinical Oncology and [2]Haematology, Department of Medicine, Leiden University Medical Centre, Leiden, The Netherlands.*

**Summary** Twenty-one identical tumour specimens were cultured both in the Plasma-Clot Diffusion Chamber (PCDC) Technique and the Human Tumour Colony-forming Assay (HTCA). The culture results achieved in the PCDC-technique were clearly superior to the HTCA: in the PCDC the mean and median plating efficiency (PE) was 0.156 and 0.147, in the HTCA 0.103 and 0.028%; adequate growth rate in the PCDC-technique was 67% and in the HTCA 38%. Fewer cells were required for plating in the PCDC-technique: $6.4 \times 10^4$ *vs.* $2.6 \times 10^5$ in the HTCA. The mean and median coefficient of variation of the colony numbers in the PCDC-technique appeared much higher: 27.3 and 37.3 *vs.* 11.2 and 11.1% in the HTCA. The relation between the PEs obtained for the same specimen in the two techniques was compared. No positive correlation was found, which can possibly be ascribed to technical shortcomings in both techniques.

Much attention has been focused on the culture of human tumours in *in vivo* and *in vitro* assays. These assays are used for drug screening and sensitivity testing of tumours of individual patients. Experiments with tumour cell lines support the hypothesis that colony formation is the most significant end point for measuring the effect of drugs on tumour cells (Roper & Drewinko, 1976; Rupniak *et al.*, 1983). The *in vitro* double layer soft-agar colony-forming assay, applied to fresh human tumours, proved promising after the first report of Hamburger & Salmon (1977). However, wider application of this technique has been hampered by many limitations, which subsequently became apparent in different studies (Selby *et al.*, 1983).

Smith *et al.* (1976) described an *in vivo* colony-forming technique using diffusion chambers implanted in mice, which seemed appropriate for the study of solid tumours. This technique has recently been modified to improve cytology of the cultured colonies (Willemze *et al.*, 1985). In this paper we report a comparative study in which samples of identical human tumours were cultured both *in vitro* and in the *in vivo* colony-forming technique.

### Materials and methods

### *Specimens*

Solid tumour specimens and effusions were obtained from 21 patients. Solid tumours were treated by mechanical and sequential enzymatic treatment (incubation for at least 2 h with 0.6% collagenase type IA and 0.002% DNAse type I – Sigma Chemical Company, St Louis, Mo., USA – at 37°C under continuous mechanical agitation). Cell suspensions were subsequently passed through 21, 23 and 25 gauge needles to eliminate cellular aggregates. Cell suspensions containing cell aggregates were not used. All specimens were cultured within 24 h after removal from the patients.

Correspondence: P.H.Th.J. Slee
Received 14 March 1985; and in revised form 9 July 1985.

### *Soft-agar colony-forming assay*

The standard technique, as described by Hamburger & Salmon (1977), was used except that DEAE-dextran, 2-mercaptoethanol, $CaCl_2$ and conditioned medium were omitted from the media and 25% cell-free ascites fluid was added per agar layer. For all specimens the same batch of ascites fluid was used which had stimulated colony growth of human ovarian cancer cell lines. The number of plated cells varied from $1 \times 10^5$–$1 \times 10^6$. The dishes were kept in a humidified incubator with 5% carbon dioxide in air at 37°C for 14–21 days until maximal growth was achieved. Colonies of >30 cells were counted using an inverted microscope.

### *Plasma-clot diffusion chamber* (*PCDC*) *technique*

Diffusion chambers were made by glueing 0.20 μm pore size, 13 mm micropore filters to one side of plastic rings and 0.20 μm nucleopore filters to the other side of the rings. Chambers were loaded with 0.1 ml cell suspension (usually $5 \times 10^4$ cells/PCDC) and 0.05 ml citrated AB plasma (Willemze *et al.* 1985). Filled chambers were kept in Hank's balanced salt solution (HBSS) at 37°C for more than 15 min until clotting occurred within the chamber. At this moment some chambers were

© The Macmillan Press Ltd., 1985

harvested to investigate the distribution of cells within the chambers. When the distribution of cells within these chambers appeared homogeneous and no more than 10 aggregates defined as groups of more than 10 cells were present, a similar single cell suspension was assumed to be present in the other implanted chambers. Under general anaesthesia two chambers were implanted in the peritoneal cavity of female, 2–3 months old, previously irradiated (8 Gy) NMRI mice. The culture time was 14–21 days; chambers were harvested every 7–10 days, as the mice did not survive longer after the irradiation, and re-implanted in new irradiated mice. After fixation and staining of the diffusion chambers as described below, colonies were counted under a binocular microscope.

*Growth characteristics*

In both techniques the single cell distribution was confirmed by a day 1 inspection to ascertain true colony growth. For the HTCA all dishes were routinely inspected on day 1, for the PCDC two or three chambers per tumour specimen were harvested and inspected. One specimen containing more than 10 aggregates per dish on day 1 was not included in this comparative study. For both techniques the same growth criteria were applied. Colonies were defined as aggregates of >30 cells. Growth was considered 'adequate', when >30 colonies were present in dishes or chambers apart from the PE. Plating efficiency (PE) is defined as number of colonies per 100 cells plated or inoculated and expressed as a percentage.

*Morphology of colonies*

In vitro *colony-forming assay* (*HTCA*): The colony-containing plate was fixed with a solution of 3% glutaraldehyde in HBSS. After the plating layer was separated from the feeder layer, it was poured gently onto a microscope slide. For even evaporation a prewetted cellulose acetate membrane was placed on top of the layer. Finally the standard Papanicolaou staining technique was applied (Salmon & Buick, 1979). Another method was to pick individual colonies from the agar with a micropipette followed by deposition on microscope slides. Fixation was carried out with a polyethylene glycol solution followed by a Papanicolaou staining.

In vivo *colony-forming assay* (*PCDC*): Diffusion chambers with a nucleopore membrane on one side and a microporous membrane on the other side were placed in a diffusion chamber holder and incubated for 60 min in a solution of 5% Ficoll (Pharmacia) and 0.5% pronase (Calbiochem, La Jolla, Ca, USA) in HBSS. The diffusion chamber holder was centrifuged to sediment all colonies onto the microporous membrane. Then the membrane was fixed in Bouin's solution and stained with giemsa or haematoxylin and eosin (Willemze *et al.*, 1985).

### Results

Fresh tumour tissue, obtained from 21 patients, (17 effusions and 4 solid specimens) was cultured using both assays. Seventeen of the tumours were derived from patients with ovarian cancer, two from breast cancers, one small cell lung cancer and one rhabdomyosarcoma. The main characteristics of all tumour specimens (with respect to tumour type, mean number of colonies ± standard deviation and PE) are given in Table I.

The number of colonies in the HTCA reached a maximum after 14–21 days, as could be observed by regular inspection. Colonies in the PCDCs could be detected after 7 days culturing, and an increase in number and size subsequently was observed according to a fairly constant growth pattern. Between days 14 and 21 the size as distinct from the number of colonies was usually increased. The HTCA resulted in adequate growth in 38% (8/21) of the specimens and the PCDC-technique in 67% (14/21). Six specimens gave rise to growth of <30 colonies per dish or chamber in both assays. Three tumours with adequate growth in both assays had a lower PE in the PCDC-technique than in the HTCA, whereas five tumours resulted in a higher PE in the PCDC-technique than in the HTCA. Seven specimens with <30 colonies per dish in the HTCA resulted in adequate growth and also a significantly higher PE in the PCDC-technique.

Summarising the culture results, the mean and median PE in the 8 specimens with adequate growth in the HTCA was 0.103 and 0.028 (range, 0.010–0.496%). In the 14 specimens with adequate growth in the PCDC-technique the mean median PE was 0.156 and 0.147 (range, 0.051–0.450%). The mean number of plated cells in the HTCA was $2.6 \times 10^5$ ($n=8$); for the PCDC-technique this was $6.4 \times 10^4$ ($n=14$). In the HTCA the number of colonies ranges from 31–892 per dish, the mean and median being 223 and 48 respectively. In the PCDC-technique the number of colonies ranged from 33–450 per chamber, the mean and median being 101 and 72 respectively. The PEs are shown in Figure 1 for all specimens with adequate growth in at least one assay: six specimens resulted in adequate growth by both techniques, whereas 10 specimens resulted in adequate growth by only one technique. The culture results obtained with the

**Table I** Culture results for identical tumour specimens in the HTCA (*in vitro* colony-forming assay) (A) and the PCDC (*in vivo* colony-forming assay) (B)

| | In vitro *colony-forming assay* (*A*) | | In vivo *colony-forming assay* (*B*) | | |
|---|---|---|---|---|---|
| *Number* | *Colonies* ±*s.d.* | *PE* | *Colonies* ±*s.d.* | *PE* | *Ratio* *B/A* |
| 1 Sov | 42 ± 0.7[a] | 0.021 | 43 ± 0[a] | 0.143 | 6.8 |
| 2 Eov | 21 ± 5.7 | 0.007 | 51 ± 30.7[a] | 0.051 | 7.3 |
| 3 Eov | 6 ± 0.7 | 0.002 | 33.5 ± 12.3[a] | 0.034 | 17 |
| 4 Eov | 41 ± 4.3[a] | 0.014 | 88 ± 32.8[a] | 0.176 | 12.6 |
| 5 Ebr | 2 | 0.002 | 450 ± 152[a] | 0.450 | 225 |
| 6 Eov | 31 ± 5.7[a] | 0.010 | 2 ± 2 | 0.001 | 0.1 |
| 7 Eov | 1 ± 0.7 | 0.001 | 171 ± 29.3[a] | 0.171 | 171 |
| 8 Eov | 8 ± 4.9 | 0.002 | 0 | | |
| 9 Sov | 6 ± 2.1 | 0.003 | 39[a] | 0.078 | 26 |
| 10 Ssclc | 2 | 0.000 | 19 ± 4.9 | 0.019 | |
| 11 Eov | 892 ± 14.6[a] | 0.496 | 20 ± 4.2 | 0.040 | 0.08 |
| 12 Srhab | 267 ± 61[a] | 0.134 | 36 ± 11.9[a] | 0.072 | 0.5 |
| 13 Eov | 0 | | 24 ± 4.6 | 0.048 | |
| 14 Eov | 22 ± 5.7 | 0.005 | 8 ± 3.4 | 0.016 | 3.2 |
| 15 Ebr | 10 ± 2 | 0.002 | 19 ± 12.7 | 0.038 | 19 |
| 16 Eov | 26 ± 7.0 | 0.009 | 76 ± 20.2[a] | 0.150 | 16.7 |
| 17 Eov | 0 | | 132 ± 34.8[a] | 0.260 | |
| 18 Eov | 425 ± 41.2[a] | 0.106 | 93 ± 2.7[a] | 0.186 | 1.8 |
| 19 Eov | 53 ± 7.0[a] | 0.035 | 97 ± 45.7[a] | 0.194 | 5.5 |
| 20 Eov | 35 ± 4.1[a] | 0.011 | 67 ± 51 | 0.134 | 12.2 |
| 21 Eov | 0 | | 43 ± 3.6[a] | 0.086 | |

ov = ovarian cancer; br = breast; sclc = small cell lung cancer; rhabd = rhabdomyosarcoma; s.d. = standard deviation in absolute numbers; ratio B/A = ratio between PE obtained with PCDC and PE with HTCA; [a] = adequate growth; E = effusion; S = solid specimen.

**Figure 1** PEs are given for identical specimens with adequate growth in one or both techniques ○ = PE for a specimen with adequate growth in only one technique; ● = PE for a specimen with adequate growth in both techniques.

PCDC-techniques are clearly superior. Differences in culture results between solid specimens and effusions cannot be given, as only 4 solid specimens and 17 effusions were cultured in both assays.

Although identical tumour material was cultured in both assays, no correlation can be given for the PEs achieved in the HTCA and the PCDC techniques.

### *Variability within two assays*

Plating in both techniques was carried out at least in duplicate, the number of experiments ranging from 2 to 11. Specimens with adequate growth were plated at least in triplicate. The coefficient of variation (CV) of the mean of the colony number in the HTCA varied from 1.7 to 22.8%, whereas the mean and median CV were 11.2 and 11.1% respectively. The CV in the PCDC-technique varied from 0 to 60.2%, whereas the mean and median CV were 27.3 and 37.3% respectively.

### *Morphology of the colonies*

The morphology of the colonies in both assays was studied to confirm that they were derived from tumour cells. For the HTCA it was almost impossible to compare the morphological characteristics of the colonies with the histology of the biopsy or the cytology of the effusion. The method described by Salmon & Buick (1979) was simple and enabled the staining of the complete agar layer, but it was often difficult to discriminate between individual cells in separate colonies. Above all, the morphology was poor. Individual colonies were also removed from the agar layer which was an arduous task. Finally only few colonies appeared to be stained. They still proved difficult to compare with the morphology of the original specimen. The morphology of the cells within the colonies derived from the PCDC-technique was clearly better and even enabled a distinction to be made between different cell types within colonies.

### Discussion

Since the work of Park *et al.* (1971) other groups have confirmed that for experimental tumours *in vitro* colony formation is the most reliable parameter for studying lethal effects induced by drugs (Thomson & Rauth, 1974; Roper & Drewinko, 1976; Courtenay, 1976).

By contrast, the *in vitro* colony-forming assays with fresh human tumour specimens have many practical and theoretical problems. The plating efficiency is low (usually one colony per 10,000 plated cells) and the number of specimens which result in adequate growth are low: in larger studies usually 40% (Von Hoff, 1983). Clumping of cells is a serious difficulty for any clonogenic cell assay, so that the descriptive term: 'colony-forming assay' is preferred to 'tumour stem cell assay' or 'clonogenic cell assay' (Agrez *et al.*, 1982, Umbach *et al.*, 1983).

The failure to grow *in vitro* may partly be explained by inadequate culture conditions. Several ways to optimize culture conditions have been investigated: the application of a low oxygen concentration, the replenishment of the medium at weekly intervals, the addition of feeder cells or cell-free ascites fluid. The addition of 25% cell-free ascites fluid has repeatedly been found to result in better growth by others (Uitendaal *et al.*, 1983), as well as ourselves (unpublished observations). Nevertheless, *in vitro* assays are frequently hampered by insufficient growth. The diffusion chamber technique may provide another way to overcome some of these problems. Tumour cells may grow better in the peritoneal cavity: nutrients, waste products and presumed humoral stimulatory and inhibitory factors can be exchanged *in vivo*, whereas the implanted cells are isolated from host cells. In this technique, tumour cell exposure to drugs is closer to the patients situation than in *in vitro* techniques.

We have compared colony growth of fresh tumour cells in an *in vivo* and an *in vitro* colony-forming assay. A significantly higher PE was seen in the *in vivo* assay than in the slightly modified *in vitro* colony-forming assay. The adequate growth rate was twice as high for the PCDC-technique as for the HTCA. The number of plated cells required for plating in the PCDC-technique was only 25% of that in the HTCA. In the PCDC-technique smaller amounts of tumour tissue can be studied and colony formation can be observed in specimens which do not result in adequate colony growth in the HTCA. A positive correlation between the PE in the PCDC-technique and in the HTCA would support the assumption that both techniques culture the same cell type, *viz.* the colony-forming cell. It could not be demonstrated in our material, although numbers were limited (Figure 1).

Hitherto all groups have used agar as a semisolid medium to immobilise the single cells in the diffusion chambers. As the agar prevents optimal morphology of the cultured colonies, plasma clots were used instead (Willemze *et al.*, 1985). Morphology in the PCDC was of good quality and enabled a comparison with the original histology or cytology. With the HTCA this was not possible.

Direct comparisons of the growth of fresh tumour cells in *in vitro* and *in vivo* colony-forming techniques have previously been reported (Courtenay *et al.*, 1978, Sobrero *et al.*, 1984). Courtenay *et al.*, (1978) described an *in vitro* technique, which is characterised by a low oxygen tension, rat red blood cells as feeder layer and test tubes (instead of Petri dishes) with a replenishable liquid phase. Although in a minority of specimens better growth was obtained in one or other of the assays, there was no evidence that either of them was superior (Courtenay *et al.*, 1978). Sobrero *et al.* (1984) compared the growth of several specimens in the colony-forming assay according to Hamburger and Salmon and the agar-diffusion chamber technique. Twenty specimens resulted in 'successful' growth in both assays. Experiments were considered 'successful', when at least 10 colonies per chamber or dish had grown. The median PE in the *in vivo* assay was more than 3 times higher than in the *in vitro* assay, although the number of plated cells in the HTCA was generally 10 times higher. Sobrero *et al.* (1984) found a positive correlation between the PE in the HTCA and the PCDC-technique for specimens with 'successful' growth. When we applied the same criteria as Sobrero *et al.* no positive correlation could be found for specimens with 'successful' growth ($n = 10$) (Figure 1 and Table I).

The coefficient of variation per colony number reflects the counting of cells, dilution, the growth of colonies and the final counting of colonies per dish or chamber. The reason for variability are partly similar for both techniques except for the growth in animals. The significant wider variation in colony numbers in the PCDC-technique may therefore be ascribed mainly to the growth in animals.

*Conclusions:*

The PCDC-technique appeared to have a higher PE and a higher growth rate for fresh tumour specimens; the number of cells inoculated in the diffusion chambers was much lower than the required number of cells to be plated in the HTCA. The (near) single cell origin of the colonies was based on inspection of all Petri dishes in the HTCA and on inspection of two or three control chambers in the PCDC-technique. The morphology of the colonies in the PCDC was superior to the morphology in the HTCA. Time and costs involved in tumour cell culture using the two techniques are more favourable in the HTCA than in the PCDC-technique. The variability in the PCDC was larger than in the HTCA possibly as a consequence of the use of animals. Drug testing in tumour bearing animals is closer to the patient situation than *in vitro* drug testing. This is even more important for drugs requiring metabolic activation. Further studies are required before a final choice can be made as to which culture technique is most appropriate for a certain application.

These studies were supported by grants LUKC R-81-2 and LUKC R-83-26 from the Koningin Wilhelmina Fonds (Netherlands Cancer Foundation). The excellent secretarial assistance of Mrs C.J.W.B. Slee-Straathof is gratefully acknowledged.

**References**

AGREZ, M.V., KOVACH, J.S. & LIEBER, M.M. (1982). Cell aggregates in the soft agar human tumour stem-cell assay. *Br. J. Cancer*, **46**, 880.

COURTENAY, V.D. (1976). A soft agar colony assay for Lewis lung tumour and B16 melanoma taken directly from the mouse. *Br. J. Cancer*, **34**, 39.

COURTENAY, V.D., SELBY, P.J., SMITH, I.E., MILLS, J. & PECKHAM, M.J. (1978). Growth of human tumor cell colonies from biopsies using two soft-agar techniques. *Br. J. Cancer*, **38**, 77.

HAMBURGER, A.W. & SALMON, S.E. (1977). Primary bioassay of human tumor stem cells. *Science*, **197**, 461.

PARK, C.H. BERGSAGEL, D.E. & McCULLOCH, E.A. (1971). Mouse myeloma tumour stem cells: A primary cell culture assay. *J. Natl Cancer Inst.*, **46**, 411.

ROPER, P.R. & DREWINKO, B. (1976). Comparison of in vitro methods to determine drug-induced cell lethality. *Cancer Res.*, **36**, 2182.

RUPNIAK, H.T., DENNIS, L.Y. & HILL, B.T. (1983). An intercomparison of *in vitro* assays for assessing cytotoxicity after a 24 hour exposure to anti-cancer drugs. *Tumori*, **69**, 37.

SALMON, S.E. & BUICK, R.N. (1979). Preparation of permanent slides of intact soft-agar colony cultures of hematopoietic and tumor stem cells. *Cancer Res.*, **39**, 1133.

SELBY, P., BUICK, R.N. & TANNOCK, I. (1983). A critical appraisal of the 'human tumor stem-cell assay'. *N. Engl. J. Med.*, **308**, 129.

SMITH, I.E., COURTENAY, V.D. & GORDON, M.Y. (1976). A colony-forming assay for human tumour xenografts using agar in diffusion chambers. *Br. J. Cancer*, **34**, 476.

SOBRERO, A. & MARSH, J.C. (1984). Chemosensitivity of human tumor clonogenic cells simultaneously assayed in agar diffusion chambers (ADC) and in a two-layer agar culture system (2-LACS). *Cancer Treat. Rep.*, **68**, 615.

THOMSON, J.E. & RAUTH, A.M. (1974). An *in vitro* assay to measure the viability of KHT tumour fibrosarcoma cells not previously exposed to culture conditions. *Radiat. Res.*, **58**, 262.

UMBACH, G., SPITZER, G., DREWINKO, B. (1983). 'Clumpogenic v Clonogenic assay' *Lancet*, **ii**, 628.

UITENDAAL, M.P., HUBERS, H.A.J.M., McVIE, J.G. & PINEDO, H.M. (1983). Human tumour clonogenicity in agar is improved by cell-free ascites fluid. *Br. J. Cancer*, **48**, 55.

WILLEMZE, R., LURVINK, E., BAKKER, W. & JOURNEE, H. (1985). An in vivo clonogenic assay for human tumours using plasma clot diffusion chambers implanted in mice. *Eur. J. Cancer Clin. Oncol.*, **21**, 127.

VON HOFF, D.D. (1983). 'Send this patient's tumor for culture and sensitivity.' *N. Engl. J. Med.*, **308**, 154.